# Evaluation of the Bioactivity of Novel Spiroisoxazoline Type Compounds against Normal and Cancer Cell Lines

**DOI:** 10.3390/molecules15129340

**Published:** 2010-12-17

**Authors:** Nigar Najim, Yaser Bathich, Mazatulikhma Mat Zain, Ahmad Sazali Hamzah, Zurina Shaameri

**Affiliations:** 1 Tissue Culture Research Laboratory, Centre of Synthesis and Chemical Biology, Institute of Science, University Technology MARA, 40450 Shah Alam, Malaysia; E-Mail: maizatul@salam.uitm.edu.my (M.M.Z.); 2 Organic Synthesis Research Laboratory, Centre of Synthesis and Chemical Biology, Institute of Science, University Technology MARA, 40450 Shah Alam, Malaysia; E-Mails: bathich@gmail.com (Y.B.); asazali@salam.uitm.edu.my (A.S.H.); zurina@salam.uitm.edu.my (Z.S.)

**Keywords:** pyrrolidine, spiroisoxazoline, cytotoxicity, neurotoxicity, neuroprotection

## Abstract

The aim of this study was to investigate the *in vitro* cellular activity of novel spiroisoxazoline type compounds against normal and cancer cell lines from lung tissue (Hs888Lu), neuron-phenotypic cells (SH-SY5Y), neuroblastoma (SH-SY5Y), human histiocytic lymphoma (U937), lung cancer (A549), and leukaemia (HL-60). Our bioassay program revealed that the spiroisoxazoline type compounds show cytotoxicity only in lymphoma cell lines, which is in contrast with the pyrrolidine precursor of these spiroisoxazoline compounds, where significant cytotoxicity is seen in all normal and cancer cell lines. These data suggest a tumour-specific mechanism of action. In addition these data also show that spiroisoxazoline compounds are non-toxic in the human neuron-phenotypic neuroblastoma SH-SY5Y cell line, and furthermore that they might protect cells from neurodegenerative disease.

## 1. Introduction

**Figure 1:**
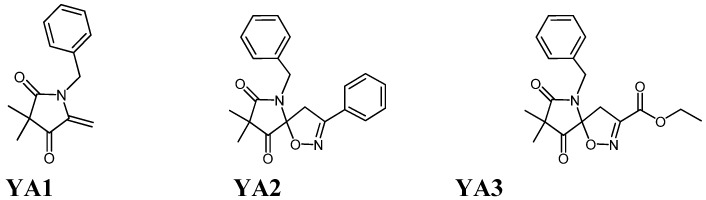
Chemical structures of the novel pyrrolidine and spiroisoxazoline type compounds.

In a continuous effort towards the synthesis of bioactive pyrrolidine type compounds [1,2,3,4], our group was able to synthesize some novel spiroisoxazoline compounds sharing a pyrrolidine and isoxazoline rings in their skeleton, and produce a variety of synthetic structures. As reported previously, natural extracts and synthetic pyrrolidine and spiroisoxazoline compounds are gaining more and more importance because of their remarkable biological properties. These recent observations have stimulated our interest in screening of the potential anticancer and neuroprotective activities of three novel compounds (see Figure 1), namely 1-benzyl-3,3-dimethyl-5-methylenepyrrolidin-2,4-dione (in this study coded as YA1), (5*S*)-6-benzyl-8,8-dimethyl-1-oxa-3-phenyl-2,6-diazaspiro{4.4}non-2-ene-7,9-dione (coded as YA2) [3] and (5*R*)-ethyl 6-benzyl-8,8-dimethyl-7,9-dioxo-1-oxa-2,6-diazaspiro{4.4}non-2-ene-3-carboxylate (coded as YA3) against a panel of normal and cancer cell lines.

Only a few publications have addressed the synthesis of this unique ring system, and none of these publications report biological activity in cancer and normal cell lines. 1β-Methylcarbapenems containing isoxazolopyrrolidine rings were synthesised and exhibited a broad antibacterial activity against both Gram-positive and Gram-negative organisms [5]. Later, in 2003 Li and co-workers synthesized the compound containing an envelope pyrrolidine ring and a planar isoxazoline ring, but again the biological activities were not reported [6]. 

Pyrrolidine is a precursor of the spiroisoxazoline compounds in this study. Pyrrolidine has been the subject of research for more than three decades. In the early 1970s, piracetam was the first pyrrolidine to come to the attention of clinicians, and since then there has been much pharmaceutical interest in agents of this type under a broad range of conditions. Modes of action of the pyrrolidines have revealed various pharmacological effects, as reviewed by Shorvon [7]. The evidence shows that the pyrrolidines exert a number of biological effects and that there is unlikely to be no single predominant mode of action that is common to all pyrrolidine compounds [7].

Natural products and synthetic compounds based on spiroisoxazoline also display a wide range of biological activities. A series of spiroisoxazoline natural products were isolated from marine sponges [8,9,10,11,12]. These spiroisoxazolines share a spiro skeleton, which consists of an isoxazoline ring and a cyclohexadiene moiety, give rise to the extensive structural diversity seen in this class of natural products [13], which exhibits various biological activities, such as antimicrobial, cytotoxic, inhibitory, and anti-inflammatory properties [13,14,15,16]. Synthetic analogues of spiroisoxazoline compounds have also been reported for their ability to inhibit the *Mycobacterium tuberculosis* detoxification enzyme mycothiol-*S*-conjugate amidase [17]. 

## 2. Results and Discussion

A survey of the literature revealed that no studies on the potential anticancer and neuroprotection activity of pyrrolidine and spiroisoxazoline type compounds had been reported, although various bioactivities have been reported previously [7,13,14,15,16,17]. Thus three novel synthetic compounds were tested and evaluated for their cytotoxic, neurotoxic, and neuroprotection ability in human normal and cancer cell lines including; normal lung tissue (Hs888Lu), differentiated (SH-SY5Y), neuroblastoma (SH-SY5Y), lymphoma (U937), leukaemia (HL-60) and lung cancer (A549). It is known that different cell lines might exhibit different sensitivities towards antiproliferative compounds, so the use of more than one cell line is therefore considered necessary in the evaluation of antiproliferative compounds and therefore a series of human cell lines of different histological origin were used in the present study. Normal lung cells are used as a model of normal cells *versus* cancer cells and this can give information about differential activity (normal *versus* tumour). Previous publications also compared normal human lung *versus* several human malignant cell lines [18,19]. 

### 2.1. Effects of novel pyrrolidine type compounds on normal and cancer cell lines

**Figure 2:**
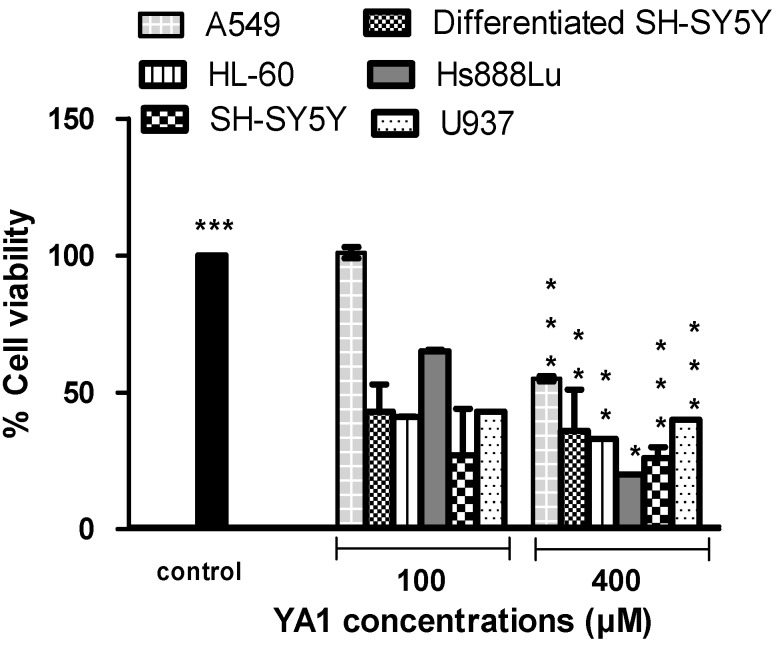
The response of normal and tumour cell lines exposed to 1-benzyl-3,3-dimethyl-5-methylenepyrrolidin-2,4-dione (YA1) for 24 h. Values are means ± SD (n=3), all comparisons were made using two-tailed Student's *t*-test (* p < 0.05, ** p < 0.01, *** p < 0.001).

Benzyl-3,3-dimethyl-5-methylenepyrrolidin-2,4-dione (YA1) showed differential cytotoxic activity against the human cell lines tested in this study (Figure 2). It exhibited a 50% reduction (*p* < 0.001) in the cell viability in HL-60, SH-SY5Y and differentiated SH-SY5Y cells compared to control after 24 h treatment at a 60 µM concentration. However, this agent was less potent in Hs888Lu and A549 cells, reducing viability by 50% reduction at 150 µM (*p* < 0.05) and > 400 µM (*p* < 0.001), respectively. The data shows that U937 is the cell line most sensitive to YA1, with an IC_50_ value of 40 μM (*p* < 0.001). The possibilities regarding mechanism of toxicity need further investigation. There have been no studies on the mechanism of action of these agents in these cancer and normal cell lines. The pyrrolidine drug class have been shown to influence cholinergic function [20,21,22,23], affect intracellular calcium and N or L type calcium channel conductance [24,25] and act as agonists at the α-aminopropionic acid (AMPA) receptor [26,27,28]. Other mechanisms of actions are increased nitric oxide synthase activity, haemorrheological and antithrombotic effects, changes to the physical properties of membranes [29] and enhanced membrane fluidity [30], presumably by binding to a membrane phospholipid. 

### 2.2. The cytotoxicity effects of novel spiroisoxazoline type compounds on normal and cancer cell lines

**Figure 3:**
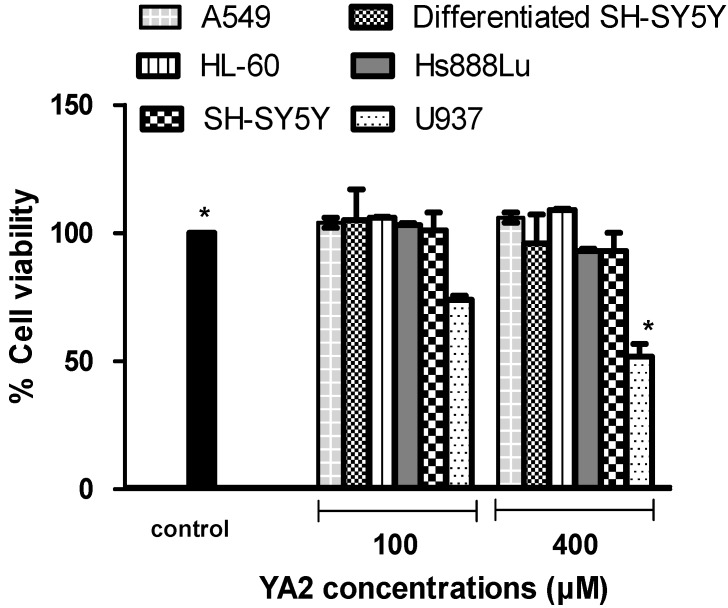
Cytotoxicity effects of YA2 in normal and tumour cell lines after 24 h incubation. Values are means ± SD (n = 3), all comparisons were made using two-tailed Student's *t*-test (* p < 0.05, ** p < 0.01, *** p < 0.001).

**Figure 4:**
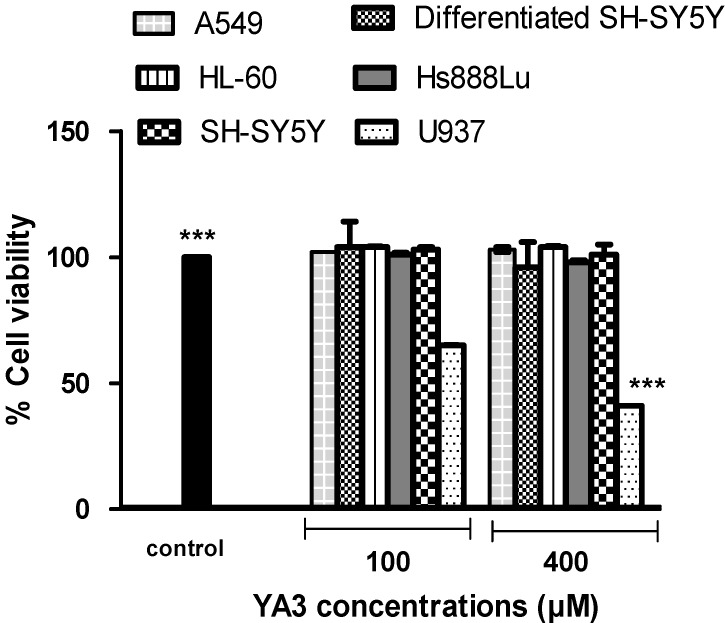
The response of normal and tumour cell lines exposed to spiroisozaxoline type compound YA3 for 24 h. Values are means ± SD (n = 3), all comparisons were made using two-tailed Student's *t*-test (* p < 0.05, ** p < 0.01, *** p < 0.001).

Incubation with (5*S*)-6-benzyl-8,8-dimethyl-1-oxa-3-phenyl-2,6-diazaspiro{4.4}non-2-ene-7,9-dione (coded as YA2) and (5*R*)-ethyl 6-benzyl-8,8-dimethyl-7,9-dioxo-1-oxa-2,6-diazaspiro{4.4}non-2-ene-3-carboxylate (coded as YA3) for 24 h clearly induced an interesting activity in the selected human cell lines, with U937 lymphoma cells showing a particularly high mortality rate after exposure to 400 μM of YA2 or YA3 for 24 h, while A549, Hs888Lu, SH-SY5Y, HL-60 and differentiated SH-SY5Y cells maintain almost 90 - 100% cell viability at concentrations up to 400 μM (Figure 3 and Figure 4). The IC_50_ value of YA2 in U937 cell lines was 119 µM after 24 h incubation, while the YA3 IC_50_ value was 92 µM under the same conditions. Some cytotoxic effect was observed at 100 μM, but the most effects were observed at 400 μM concentration. When drugs are evaluated for antitumor action *in vitro*, it may be more desirable to compare their cytotoxic action on malignant cells with that on normal cells. Ideally a compound should show differential activity in tumour cells compared to normal cells. Our novel finding suggests that compounds YA2 and YA3 show a tumour specific mechanism of action against lymphoma U937 compared to other normal and cancer cell lines.

### 2.3. The neurotoxicity and neuroprotection effects of novel spiroisoxazoline type compounds

**Figure 5:**
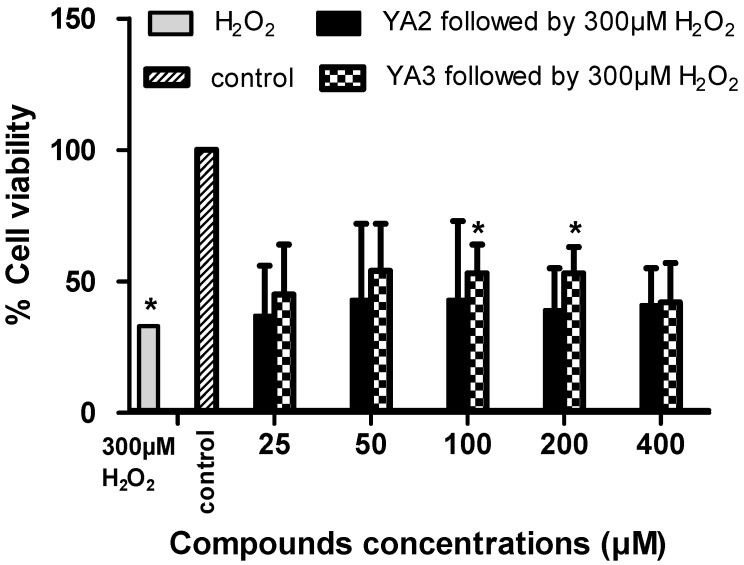
Neuroprotective activity of synthetic spiroisoxazoline type compounds. Differentiated SH-SY5Y cells were treated with 300 µM hydrogen peroxide in the presence or absence of various concentrations of YA2 or YA3. Twenty-four hours later, cell viability was determined by MTS assay. Data are presented as mean ± SD from triplicate samples, all comparisons were made using two-tailed Student's *t*-test (* p < 0.05, ** p < 0.01, *** p < 0.001).

Our data have shown that YA2 and YA3 at concentrations ≤ 400 µM have no effect on neuron phenotypic cells (differentiated SH-SY5Y; Figure 3 and Figure 4). We therefore investigated whether YA2 and YA3 can exert a neuroprotective effect on the neuron phenotypic cells, SH-SY5Y. Hydrogen peroxide induced neurotoxicity was used as a positive control. Incubation of the differentiated cells in 300 µM of H_2_O_2_ resulted in a drastic decrease (67%) in cell viability (Figure 5). This is in agreement with earlier observations that H_2_O_2_ was toxic and increased cell loss as well as the number of apoptotic cells [31]. Incubation with YA2 or YA3 for 2 h followed by exposure to 300 µM H_2_O_2_ showed almost 15% increase in cell viability compared to the cells incubated with 300 µM H_2_O_2_ only (Figure 5). YA3 exhibited a significant (*p* < 0.05) increase in cell viability at concentrations 100 and 200 µM. YA2 did not show significant difference from control. These findings are particularly relevant, since H_2_O_2_ is quantitatively the most important of the peroxides generated in brain cells [32], and its intracellular accumulation can induce oxidative stress leading to neuronal apoptosis [33]. These results led us to consider that YA3 may be involved in the neuroprotective effect seen in the SH-SY5Y cell lines. Our studies are the first to demonstrate the cytotoxicity, neurotoxicity and neuroprotection effects of spiroisoxazolines type compounds in culture cell lines.

### 2.4. Effects of spiroisoxazoline type compounds on cell cycle and apoptosis levels

**Figure 6:**
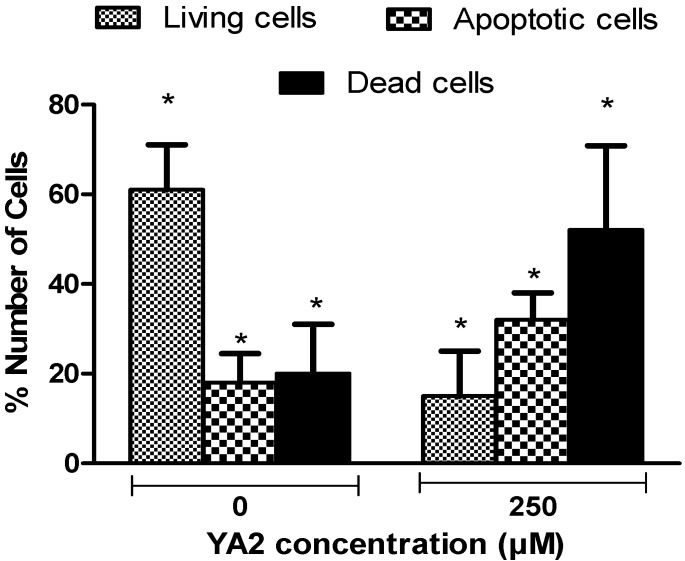
Shows the results expressed as percentage of living, apoptotic, and dead cells in U937 cell lines treated with YA2 for 24h. Values are means ± S.D. of at least three independent experiments (* *P* < 0.05, ** *P* < 0.01, *** *P* < 0.001). All comparisons were made using two-tailed student’s *t*-test.

To show whether the growth inhibition induced by spiroisoxazoline type compounds in U937 cells was caused by induction of apoptosis, the cells were stained with Annexin V-Biotin. Apoptotic rate was analyzed by bioanalyzer [34], as shown in Figure 6. The apoptotic cell population in U937 cell lines increased gradually from 18% in control, to 32% after exposure to 250 µM YA2 for 24 h. Significant decreases in living and increases in necrotic cell population (p < 0.05) were observed following 24 h of treatment with 250 µM YA2 (Figure 6). Treatment with YA3 also exhibit significant increase in apoptotic cell population under the same conditions (data not shown). 

Apoptosis play an important role in the development and maintenance of homeostasis with all multicellular organisms [35] and activation of apoptosis pathways is now recognized to be a key mechanism by which cytotoxic drugs kill tumour cells [36]. Previous studies indicate that apoptosis pathways can now be considered a significant index for the selection of new anti-tumour drugs [36,37,38]. In this study, our results demonstrate that YA2 and YA3 can elicit significant cytotoxic effects by induction of apoptosis on lymphoma U937 cells. The data suggest that these compounds may be used as an effective apoptosis inducer on lymphoma cells *in vitro*. However, these possibilities need further investigation in the context of the present experimental conditions. Therefore, decrease in U937 cell viability and an increase in apoptosis cells might result from the altering the balance of Bcl-2 and Bax protein expression and activation of the caspase-3 pathway [39,40]. Previous studies have demonstrated that the induction of apoptosis in U937 cells was associated with p38 MAP kinase cascade activation [41]. 

**Figure 7:**
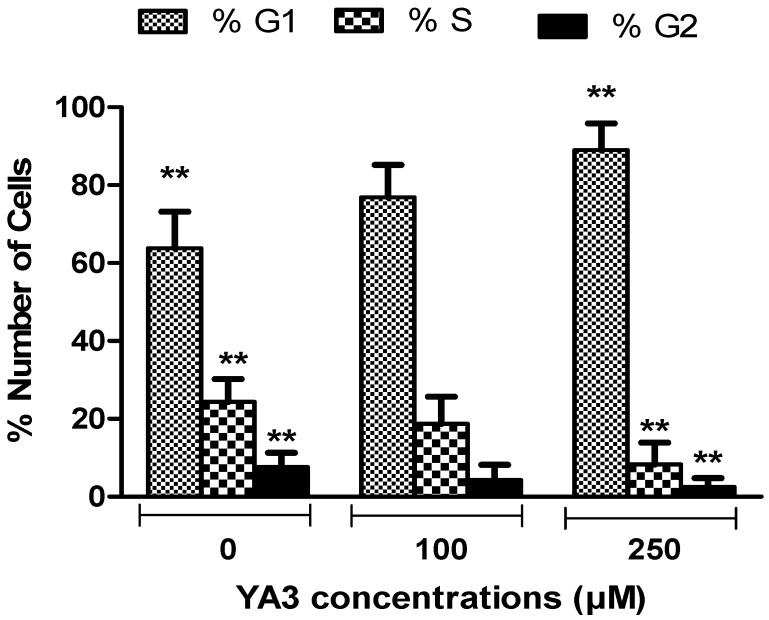
Cell cycle analysis of U937 cell lines treated with various concentrations of YA3 for 24h. Results are expressed as percentage of cells in G1, S, and G2/M phases of the cell cycle. The results of all treatments experiments were compared to control. The results for each time point represent the mean ± standard deviation of three separate experiments (* *P* < 0.05, ** *P* < 0.01, *** *P* < 0.001). All comparisons were made using two-tailed student’s *t*-test.

In order to determine whether the reduction in cell number was related to cell cycle arrest, we quantified cell cycle distribution using flow cytometry, as shown in Figure 7. Almost 63% of the untreated control cells were in the G1 phase. In contrast, treatment with 250 μM of YA2 or YA3 resulted in a significantly greater proportion of cell in the G1 phase {85% (*p* < 0.01) and 89% (*p* < 0.05) respectively}. The increased number of cells in the G1 phase following spiroisoxazoline exposure was related to a decrease in the S and G2/M phase, relative to control cells. The pathways specific to these effects are under investigation. Previous studies demonstrated that the G0/G1 block in U937 can be due to different effects such as; decrease in cyclin E and increase in cyclin D and p27 [42]. Dong Moon [43] found that G1 arrest through up-regulation of cyclin-dependent kinase (Cdk) inhibitor, produces morphological features of apoptosis in U937 cells. Later studies have demonstrated that G0/G1 growth arrest in U937 cells was accompanied by an increase in VCP/p97 expression [44].

## 3. Experimental

### 3.1. Chemical Compounds

Three novel compounds were tested in this study; pyrrolidine 1-benzyl-3,3-dimethyl-5-methylenepyrrolidin-2,4-dione (YA1) and spiroisoxazoline type compounds; (5*S*)-6-benzyl-8,8-dimethyl-1-oxa-3-phenyl-2,6-diazaspiro{4.4}non-2-ene-7,9-dione (YA2) and (5*R*)-ethyl 6-benzyl-8,8-dimethyl-7,9-dioxo-1-oxa-2,6-diazaspiro{4.4}non-2-ene-3-carboxylate (YA3). The two spiroisoxazoline compounds (YA2 and YA3) were synthesized from the pyrrolidine 1-benzyl-3,3-dimethyl-5-methylenepyrrolidin-2,4-dione (YA1). 

### 3.2. Cell lines and culture conditions

Human neuroblastoma SH-SY5Y cell lines were a gift from Dr. Carol Sanfeliu (Department of Pharmacology and Toxicology, Institute of Biological Research, Barcelona, Spain). Lymphoma U937 and lung cancer A549 cell lines were a gift from Dr. Mohamed Saifulaman (Faculty of Applied Sciences, UiTM, Malaysia). Normal lung Hs888Lu cell lines were purchased from American Type Culture Collection (ATCC, The Global Bioresource Centre, Manassas, VA, USA). A leukaemia (HL-60) cell was a gift from Mr. Mohd. Hafiz Bin Mohd. Rothi (Faculty of Applied Sciences, UiTM, Malaysia). 

The SH-SY5Y, U937 and A549 cells were cultured in Dulbecco’s Modified Eagle’s Medium (DMEM, Sigma, USA) with high glucose content, 1% non-essential amino acids (100 ×) (PAA Laboratory GmbH, Austria), 1% L-glutamine (200 mM) (Sigma, USA), 1% gentamicin (10 mg/mL) (PAA Laboratory GmbH, Austria), and supplemented with 10% fetal bovine serum (FBS, PAA Laboratory GmbH, Austria). For treatment experiments neuroblastoma (SH-SY5Y) cells were adapted to grow in 1:1 of Minimum Essential Medium Eagle: Nutrient mixture F12-Ham (EMEM : Hams-F12, Sigma, USA) with 1% non-essential amino acids, 1% L-glutamine, 1% gentamicin and supplemented with 10% FBS. HL-60 cultured in Isove's Modified Dulbecco’s Medium (IMDM, Sigma, USA) supplemented with 20% FBS. Hs888Lu was adapted to grow in DMEM with high glucose content, 1% non-essential amino acids, 2% L-glutamine (200 mM), 1% penicillin/streptomycin (100 ×) (PAA Laboratories GmbH, Austria), 1% sodium pyruvate (1 mM) (Sigma-Aldrich, USA), and supplemented with 10% fetal bovine serum (FBS). All cell lines were maintained in an incubator (Contherm Scientific Ltd, NEW ZELAND) at 37ºC in a 5% CO_2_ atmosphere with 95% humidity. 

Culture conditions were optimized for each cell line. Each cells was tested for the solvent dimethyl sulfoxide (DMSO, Sigma, USA) activity at various DMSO percentage ranging from 0.25 – 4% and under the same culture conditions. The final DMSO concentration in the test was < 0.5%. The compounds reconstituted in DMSO with stock concentration (100 mM) and the stock solutions were stored as aliquots at -20 ºC until required. For initial screening, each compound was routinely tested at various dilutions. The stock concentrations were then diluted with complete medium prior each experiment, resulting in a series of final concentrations ranging from 1nM to 400 µM. The cytotoxicity, neurotoxicity, neuroprotection of synthetic novel pyrrolidine, spiroisoxazolines type compounds and DMSO activity were performed by CellTiter 96^®^ AQ_ueous_ Assay uses the novel tetrazolium compound (3-(4,5-dimethylthiazol-2-yl)-5-(3-carboxymethoxyphenyl)-2-(4-sulfophenyl)-2H-tetrazolium, inner salt; MTS, Promega, USA) using Glomax multi detection system (Promega, USA) and read at 490 nm [45]. 

### 3.3. Assay for cytotoxic activity

Cells (1×10^5^ cells/ml) were seeded in 96-well plates and left to grow overnight in humidified atmosphere containing 5% CO_2_ at 37 ºC. The following days, cells were treated with serial dilution of novel compounds (1 nM – 400 µM) in triplicates. After 24 h cell viability was measured using the MTS assay. Results were representative of at least three independent experiments, and were expressed as percentage of the value observed with no drug treatment (control). 

### 3.4. Neurotoxicity and neuroprotection activities

Retinoic acid (RA, Sigma, USA) will induce the differentiation of the neuroblastoma cells to behave like neuron-phenotypic cells [46,47]. Approximately, 1×10^4^ cells per well were seeded in 96-well plate. After 24 h, RA was added at a final concentration of 10µM in complete EMEM-F12 media. The medium in plate was changed at day 3 with fresh RA and cultures were ready to be tested at day 6. The synthetic spiroisoxazolines type compounds; YA2 and YA3 were tested for their neurotoxicity and neuroprotection effects. The compounds serial dilutions in MEME-F12 were made fresh prior to each test. For neurotoxicity; the differentiated cells in each well were tested with final concentrations of compounds ranging from 1 nM to 400 µM. The wells were agitated lightly and incubated for 24 h. On the next day, treated cultures were tested for cell viability using MTS assay. In case of neuroprotection; the cultures were incubated with a serial dilution of compounds at final concentrations ranging from 1 nM to 400 µM for 2 h. Cells were subsequently exposed to 300 µM hydrogen peroxide (H_2_O_2_, 30%, MERCK, Germany, which caused 33% of cell viability). The cultures were further incubated for 24 h, and then cell viability test was performed. 

### 3.5. Cell cycle phase distribution

Cells in exponential growth were counted and plated in 6-well plates at a cell density 1×10^5^ cells/mL in medium for 24 h. The cells were then treated with 10, 100, and 250 µM of YA2 or YA3 for 24 h. Following the treatments, cells were harvested according to the method of Kalejta *et al*. [48] and analysed using flow cytometry (FACSCalibur, BD Bioscience) as described previously [48]. Quantification of the cell cycle distribution and the percentage of the distinct cell cycle phases were gated and calculated using the FCSexpress and MultiCycle software.

### 3.6. Apoptosis assay

Following the treatments as described earlier, 0.5 mL of cell suspension was transferred from 6 well plates to a microfuge tube, and centrifuged for 5 minutes at 1,000×g. The cells then washed with 0.5 mL phosphate buffer saline (PBS, 0.01M, Sigma, USA) and centrifuged for another 5 minutes. The cells were then resuspended in 0.5 mL cold 1× Binding buffer and 1.25 µL Annexin V- Biotin (Annexin V-Biotin apoptosis detection kit, Calbiochem^®^, U.S.) and incubated 15 minutes in dark at room temperature. Following incubation the cells centrifuged for 5 minutes at 1,000×g and cells resuspended in 0.5 mL 1× Binding buffer, 1 µL Cy^TM^5-streptavidin (1 mg/ 1 mL deionised water, GE Healthcare, UK) and 50 µL calcein-AM solution (final concentration 0.5 µL, Fluka, Swizerland) were added. The samples were incubated for another 15 minutes away from light and the apoptotic cells detected using bioanalyzer (Agilent 2100 Bioanalyzer, Germany). 

### 3.7. Statistical analysis

All determinations were performed at least in triplicate, means and standard deviations were determined. All comparisons were made using two-tailed Student’s *t*-test, and when positive indicated by asterisks (* *p* < 0.05, ** *p* < 0.01, *** *p* < 0.001) using GraphPad PRISM^®^ version 5.0. 

## 4. Conclusions

In summary, the results clearly demonstrated a selective toxicity of synthetic spiroisoxazolines YA2 and YA3 against the human lymphoma U937 cell line. In contrast no similar effects were observed against neuroblastoma (SH-SY5Y), lung cancer (A549), leukaemia (HL-60), neuron phenotypic cells (differentiated SH-SY5Y), and normal lung (Hs888Lu) cell lines exposed to concentrations of synthetic spiroisoxazoline up to 400 µM for 24 h. The results shown that YA2 and YA3 are not toxic to the neuron cells, and YA3 is able to protect against cell loss induced by H_2_O_2_. Thus this compound may have a role in the prevention of neurodegenerative disease. Compound YA1 showed a different cytotoxic pattern against the human cell lines tested in this study. YA1 is more toxic to U937 and HL-60 cells. However, its toxicity in Hs888Lu and SH-SY5Y was significantly less under the same conditions. Since the compounds YA2 and YA3 are toxic only in U937 cells with IC_50_ values 119 µM and 92 µM respectively, then this is very interesting as it may suggest a tumour specific mechanism of action. Further studies are required to establish in detail the possible mechanism of actions and potential side effects of these compounds. However the evidence of both differential cytotoxicity combined with the potential for neuroprotection makes this an interesting class of molecules for further research and development.
